# Lack of Clinical Relevance of ANA and ASMA Positivity in Patients with Liver Transplantation without a History of Autoimmune Diseases

**DOI:** 10.1155/2017/2456916

**Published:** 2017-02-27

**Authors:** Lucienne Pellegrini, Gianpaolo Parrilli, Antonella Santonicola, Luigi Cinquanta, Cesare Caputo, Carolina Ciacci, Fabiana Zingone

**Affiliations:** ^1^AOU San Giovanni di Dio e Ruggi d'Aragona, Liver Following Transplantation Centre, Department of Medicine and Surgery, University of Salerno, Salerno, Italy; ^2^AOU San Giovanni di Dio e Ruggi d'Aragona, UOC Clinical Pathology Department, Salerno, Italy

## Abstract

The relevance of isolated autoimmunity elevation in orthotopic liver transplantation (OLT) patients is unknown. Our aim was to analyse how serum autoantibodies change in time and to evaluate their clinical relevance in OLT patients. Patients were invited to provide samples to evaluate ANA, AMA, ASMA, and LKM at the time of enrolment (*T*0), after 6 months (*T*6), and after 12 months (*T*12). We included 114 patients in the study (76% males, median age 62.5 years), finding isolated elevation of at least one serum antibody in up to 80% of them. We described fluctuating positive autoantibodies in the one year of observation, with only 45.6% of patients positive for ANA and less than 2% positive for ASMA, at all three times. Isolated elevation of tissue antibodies was not related to gender, age, HCC at transplant, early rejection, cause of transplantation, immunotherapy taken, and age at the time of the study. We did not detect a higher prevalence of positive autoimmunity in patients with signs of liver injury. ANA and ASMA evaluation in patients with liver transplantation and no history of autoimmune disease has no clinical relevance, since it varies in time and is not related to any risk factors or liver injury. Routine autoimmunity evaluation should be avoided.

## 1. Introduction

Isolated elevation of serum autoantibodies such as antinuclear antibodies (ANA), anti-smooth muscle antibodies (ASMA), anti-liver-kidney microsomal antibodies (anti-LKM), and antimitochondrial antibodies (AMA) has been described in subjects with orthotopic liver transplantation (OLT) without a history of prior autoimmune liver disease [1–5]. This isolated elevation is insufficient for the diagnosis of de novo autoimmune disease [6, 7]; therefore we know little of its clinical relevance.

In the existing literature, the prevalence of isolated elevation of serum autoantibodies in OLT patients varies. This variability may relate to demographic factors, cause of liver transplantation, difference in the cut-off points above which it is considered positive, time passing since liver transplant at test time, or immunotherapy used. Chen et al. [2] detected positivity of one or more autoantibodies post-OLT (cut-off ≥ 1 : 40 for ANA and/or ≥1 : 20 for ASMA, anti-LKM, and AMA) in 51 children out of a population of 68 (75.0%). The present study considers immunosuppressive treatment with cyclosporine, multiple episodes of rejection, and abnormal ALT, as risk factors for the development of autoantibodies. Recently, Foschi et al. [8] have tested the serum of 92 patients who had undergone transplantation, finding that 64% (cut-off ≥ 1 : 80) of them had positive autoimmunity. According to the authors, gender, cause of transplantation, and immunotherapy (cyclosporine versus tacrolimus) were not associated with autoimmune positivity, while they found a correlation with liver injury. Several studies point to the role of serum autoantibodies as predictors of graft dysfunction [2, 3, 5, 9]. However, to the best of our knowledge, no study discusses the variability of these autoantibodies over time and the fact that they might continue to show change for months if not a year, suggesting that their pathological interpretation is likely to be of little significance.

Our aim was to analyse how the autoantibodies values change in the span of one year in subjects with OLT. We also looked at the possible association of their positivity with several risk factors to evaluate its clinical relevance in this population.

## 2. Material and Methods

The study population consisted of patients with OLT recruited from the Liver Following Transplantation Centre of the University of Salerno, who were prospectively followed from May the 1, 2015, to May 31, 2016. The inclusion criteria of the study cohort were written informed consent and age more than 18 years at the time of inclusion. We excluded all patients who had undergone transplantation for autoimmune hepatitis or cholangitis autoimmune diseases (primary biliary cholangitis or primary sclerosing cholangitis), patients with any other autoimmune disease (autoimmune thyroiditis, coeliac disease, Systemic Lupus Erythematosus, etc.), and those with not specific positivity for autoimmune markers (ANA, ASMA, AMA, and LKM) before liver transplantation. We excluded patients who had undergone transplantation less than 3 months prior to recruitment and any patients who were undergoing therapy modification during the study.

The patients were invited to provide samples for the evaluation of the autoantibodies ANA, AMA, ASMA, and LKM at the enrolment (*T*0), after 6 months (*T*6), and after 12 months (*T*12). The antibodies were detected by indirect immunofluorescence using Hep-2 cells for ANA as highly sensitive substrates and rodent tissue sections (triple tissue testing) for AMA, ASMA, and LKM (Delta Biologicals Srl, Pomezia, Italy). The analyses, at the 3 time points, were performed at the same time in parallel on the same slides. A positive test for ANA was defined as titers ≥1 : 80 and for AMA, ASMA, and LKM ≥1 : 40 [10, 11]. These analyses were performed in addition to the patients' routine laboratory follow-up, which included liver injury tests (ALT and GGT: considering a value of >40 and >50 UI/mL pathological, resp.), immunoglobulins isotype G, serum protein electrophoresis, C-reactive protein (CRP) (normal values < 0.5 mg/dL), and CMV and EBV IgG and IgM.

We referred to the existing literature [2, 8] to investigate several possible risk factors of positive autoimmunity tests: gender; cause of transplantation (cirrhosis related to HBV, HCV, or other chronic liver diseases such as alcoholic cirrhosis, polycystic liver disease, congenital fibrosis, Wilson disease, and nonalcoholic liver disease); HCC at the time of transplantation; early liver rejection; time from transplantation (time from the date of liver transplant to the date of the first evaluation of our analysis (*T*0)); patient's age at the first evaluation of our analysis (*T*0); therapy at the time of the study which remained unchanged through the whole study period.

## 3. Statistical Analysis

Categorical and continuous variables were expressed as frequency and median (range), respectively. Differences in frequencies were calculated using *χ*^2^ test. Univariate logistic analysis was used to test the risk of positive autoimmunity in subjects with pathological ALT and GGT values, compared to those with normal values. All analyses were performed using Stata version 12, Stata Corp., College Station, TX.

## 4. Results

At the time of enrolment (May 2015) 123 patients were followed at our Liver Following Transplantation Centre of the University of Salerno. Three patients were excluded because they had undergone transplantation two months prior to enrolment, two because they had shown ANA positivity before the liver transplantation, and 4 because they had undergone liver transplantation for autoimmune liver diseases. Therefore, the study population consisted of 114 patients (76% males, age at *T*0 62.5 (range 24.3–77.2)) who underwent blood tests at time *T*0 (May 2015), at *T*6 (October 2015), and at *T*12 (May 2016).  Table 1 summarises the characteristics of the study population.

We reported AMA positivity in one patient at all three times (1 : 320 at *T*0, 1 : 160 at *T*6, and 1 : 80 at *T*12). LKM were never positive. Conversely, looking at ANA and ASMA we noted a fluctuation of the results in one-year time. ANA showed positivity in 79.8% of patients at *T*0, in 70.2% at *T*6, and in 65.8% after one year (*T*12). ASMA showed positivity in 11.4% of patients at *T*0, 16.7% at *T*6, and 16.7% at *T*12.

At no time was ANA positivity related to gender, cause of transplantation, HCC at the time of transplantation, early liver rejection, time from transplantation, and age at the time of the study. At *T*0 we observed a lower percentage of ANA positivity in patients treated with everolimus (7/15), compared to subjects treated with other immunotherapies. However, this result was not confirmed at *T*6 and *T*12 (Table 2). Similarly, ASMA were related to none of the above risk factors during the study period, but we did find a higher percentage of ASMA positivity in patients with HCV (11/57) and in those with HCC at the time of transplantation (8/40) at *T*0. However, these results were not confirmed at *T*6 and *T*12 (Table 3).

Patients showing pathological values of ALT and GGT have no statistically significant higher risk of ANA and ASMA positivity at any time* (data not shown)*. We only observed an increased risk of ANA positivity in patients with GGT > 40 at *T*6 (OR 2.51, 95% 1.04–6.05) that was not present at *T*0 and *T*12.

All subjects had CMV and EBV IgG antibodies and they tested negative for CMV and EBV IgM at all times. No patient met the criteria of autoimmunity liver disease.

Fifty-two patients (45.6%) had positive ANA (≥1 : 80) at all three times; only two had positive ASMA at all three times. The AMA positive patient was ANA and ASMA negative.

Conversely, eleven patients had negative ANA (<1 : 80) at all three times; seventy-eight tested negative to ASMA at all three times (Table 4).

Table 4 shows that the patients with positive and negative ANA or ASMA at all three times were distributed in all subgroups. Also, we can detect no factors influencing the autoantibodies' results through this distribution.

## 5. Discussion

Isolated elevation of at least one tissue antibody existed in up to 80% of our study population. However, we described fluctuating positive autoimmunity during the one year of observation, with 45.6% ANA positive patients and less than 2% ASMA positive at all three times. Isolated elevation of tissue antibodies was not related to gender, age, HCC at transplant, early rejection, cause of transplantation, immunotherapy taken, and age over the year of observation. Moreover, no higher prevalence of positive autoimmunity in patients with signs of liver injury was reported.

The literature reports that ANA positivity is described in approximately 25% of the general population, it is higher in females than in males, and it is often unspecified and not related to age [12, 13]. In fact, it is known that most individuals with, for example, a positive ANA do not have an autoimmune disease and most of them will not develop one. However, in accordance with our study, the prevalence of positive autoimmunity seems higher in subjects who have undergone liver transplantation. In particular, it has been reported in 75% of children [2] and 64% of adults [8].

Why some liver transplant recipients tend to form serum autoantibodies after transplant is unclear. On this matter, some papers suggest that autoantibodies may form in response to the stimulation induced by autoantigens released from damaged tissue [3, 5, 9]. Other studies point to the role of polyclonal stimulation through viral infection. Infection with CMV is suggested as a possible trigger for autoimmunity, due to the presence of CMV IgM in the sera of patients with de novo autoimmune disease [14]. Finally, the use of calcineurin inhibitors may interfere with T-lymphocytes maturation, thus inducing autoimmunity [3, 4, 7]. Our findings did not support any of these possible, valid explanations. Rather, the strength of our study is that the autoantibodies are evaluated three times in the course of one year, in the same laboratory, and in the same population. We can thus appreciate their fluctuation and the absence of a constant association with any risk factors, independent from sex, therapy taken, and any other values that may be taken into consideration, even in those people where antibodies are positive at all time.

We cannot exclude that in some patients the constant and continued evidence of increased tissue autoantibodies plays a direct role in the pathogenesis of further autoimmune diseases [15]. In most cases, however, they just may be considered an epiphenomenon of the disease and have no role in it [16]. Moreover, even when these autoantibodies play a role in the emergence of autoimmune diseases, the effector mechanisms necessary for the development of clinical disease may be impaired in older subjects, which make up most of the transplanted population. In fact, old age may determine that a longer period of time incurred between antibody positivity and clinically apparent joint disease [17]. Finally, in older subjects the mechanisms and the meaning of autoimmunity may be only a reflection of the advanced organ damage caused by aging and the resulting immune response [18].

We can therefore conclude that ANA and ASMA evaluation in patients with liver transplantation and no history of autoimmune disease has no clinical relevance since it varies in time and is not related to any risk factors or liver injury. Since it is of critical importance to improve cost containment and overall health economics management, routine autoimmunity evaluation should be avoided.

## Figures and Tables

**Table 1:** Study population characteristics (114 patients).

Variables	
Median age at the time of transplantation (range)	53 years (1–67)
*N* of males (%)	76 (66.7)
Cause of transplantation *N* (%)	
(i) HBV	39 (34.2)
(ii) HCV	57 (50)
(iii) Other^*∗*^	18 (15.8)
HCC at the time of transplantation *N* (%)	40 (35)
Early liver rejection *N* (%)	27 (23.7)
Time from transplantation (*T*0-time of the transplant) *N* (%)	
(i) >3 months–1 year	11 (9.6)
(ii) >1 year–5 years	27 (23.7)
(iii) >5 years–10 years	26 (22.8)
(iv) >10 years	50 (43.9)
Age group at *T*0	
(i) ≤50 years	15 (13.2)
(ii) >50–60 years	34 (29.8)
(iii) >60 years	65 (57)
Therapy at the time of the study (constant during the study) *N* (%)	
(i) Cyclosporine	32 (28.1)
(ii) Tacrolimus	55 (48.2)
(iii) Mycophenolate	12 (10.5)
(iv) Everolimus	15 (13.2)

^*∗*^10: alcoholic cirrhosis, 2: polycystic liver disease, 1: congenital fibrosis, 1: Wilson's disease, and 4: nonalcoholic liver disease.

**Table 2:** ANA positivity (≥1 : 80) according to different risk factors during the study period.

		ANA positivity (≥1 : 80)		
	Total number of patients	*T*0	*T*6	*T*12
Gender				
(i) Male	**76**	59 (77.6)	52 (68.4)	47 (61.8)
(ii) Female	**38**	32 (84.2)	28 (73.7)	28 (73.7)
		*P* 0.4	*P* 0.5	*P* 0.2
Cause of transplantation				
(i) HBV	**39**	31 (79.5)	26 (66.7)	23 (59)
(ii) HCV	**57**	45 (78.9)	42 (73.7)	39 (68.4)
(iii) Other	**18**	15 (83.3)	12 (66.7)	13 (72.2)
		*P* 0.9	*P* 0.7	*P* 0.5
HCC at the time of transplantation				
(i) No	**74**	58 (78.4)	56 (75.7)	50 (67.6)
(ii) Yes	**40**	33 (82.5)	24 (60)	25 (62.5)
		*P* 0.6	*P* 0.08	*P* 0.6
Early rejection				
(i) No	**87**	69 (79.3)	59 (67.8)	56 (64.4)
(ii) Yes	**27**	22 (81.5)	21 (77.8)	19 (70.4)
		*P* 0.8	*P* 0.3	*P* 0.5
Time from transplantation				
(i) >3 months–1 year	**11**	9 (81.8)	7 (63.6)	10 (90.9)
(ii) >1 year–5 years	**27**	20 (74.1)	19 (70.4)	14 (51.8)
(iii) >5 years–10 years	**26**	20 (76.9)	19 (73.1)	17 (65.4)
(iv) >10 years	**50**	42 (84)	35 (70)	34 (68)
		*P* 0.7	*P* 0.9	*P* 0.1
Age group at *T*0				
(i) ≤50 years	**15**	11 (73.3)	11 (73.3)	12 (80)
(ii) >50–60 years	**34**	27 (79.4)	24 (70.6)	20 (58.8)
(iii) >60 years	**65**	53 (81.5)	45 (69.2)	43 (66.1)
		*P* 0.7	*P* 0.9	*P* 0.3
Therapy at the time of the study (constant during the study)				
(i) Cyclosporine	**32**	28 (87.5)	23 (71.9)	21 (65.6)
(ii) FK	**55**	46 (83.6)	39 (70.9)	38 (69.1)
(iii) Mycophenolate	**12**	10 (83.3)	7 (58.3)	8 (66.7)
(iv) Everolimus	**15**	7 (46.7)	11 (73.3)	8 (53.3)
		*P* 0.007	*P* 0.8	*P* 0.7

**Table 3:** ASMA positivity (≥1 : 40) according to different risk factors during the study period.

		ASMA positivity (≥1 : 40)		
	Total number of patients	*T*0	*T*6	*T*12
Gender				
(i) Male	**76**	9 (11.8)	12 (15.8)	15 (19.7)
(ii) Female	**38**	4 (10.5)	7 (18.4)	4 (10.5)
		*P* 0.8	*P* 0.7	*P* 0.2
Cause of transplantation				
(i) HBV	**39**	1 (2.5)	5 (12.8)	3 (7.7)
(ii) HCV	**57**	11 (19.3)	10 (17.5)	11 (19.3)
(iii) Other	**18**	1 (5.5)	4 (22.2)	5 (27.8)
		*P* 0.03	*P* 0.6	*P* 0.1
HCC at the time of transplantation				
(i) No	**74**	5 (6.7)	13 (17.6)	12 (16.2)
(ii) Yes	**40**	8 (20)	6 (15)	7 (17.5)
		*P* 0.03	*P* 0.7	*P* 0.8
Early rejection				
(i) No	**87**	12 (13.8)	12 (13.8)	13 (14.9)
(ii) Yes	**27**	1 (3.7)	7 (25.9)	6 (22.2)
		*P* 0.1	*P* 0.1	*P* 0.4
Time from transplantation				
(i) >3 months–1 year	**11**	1 (9.1)	2 (18.2)	1 (9.1)
(ii) >1 year–5 years	**27**	5 (18.5)	7 (25.9)	7 (25.9)
(iii) >5 years–10 years	**26**	3 (11.5)	2 (7.7)	3 (11.5)
(iv) >10 years	**50**	4 (8)	8 (16)	8 (16)
		*P* 0.5	*P* 0.3	*P* 0.4
Age group at *T*0				
(i) ≤50 years	**15**	1 (6.7)	3 (20)	1 (6.7)
(ii) >50–60 years	**34**	4 (11.7)	3 (8.8)	4 (11.7)
(iii) >60 years	**65**	8 (12.3)	13 (20)	14 (21.5)
		*P* 0.8	*P* 0.3	*P* 0.2
Therapy at the time of the study (constant at *T*0-*T*6 and *T*12)				
(i) Cyclosporine	**32**	2 (6.2)	4 (12.5)	7 (21.9)
(ii) FK	**55**	8 (14.5)	10 (18.2)	8 (14.5)
(iii) Mycophenolate	**12**	1 (8.3)	1 (8.3)	0 (0)
(iv) Everolimus	**15**	2 (13.3)	4 (26.7)	4 (26.7)
		*P* 0.6	*P* 0.5	*P* 0.2

**Table 4:** Characteristics of patients with ANA and ASMA positive and negative at all three times.

Variables	Total number of patients 114	ANA always positive 52	ANA always negative 11	ASMA always positive 2	ASMA always negative 78
Gender (%)					
(i) Male	**76**	32 (42.1)	8 (10.5)	2 (2.6)	52 (68.4)
(ii) Female	**38**	20 (52.6)	3 (7.9)	0 (0)	26 (68.4)
		*P* 0.2	*P* 0.6	*P* 0.3	*P* 1.0
Cause of transplantation					
(i) HBV	**39**	13 (33.3)	3 (7.7)	0	32 (82.0)
(ii) HCV	**57**	30 (52.6)	6 (10.5)	2 (3.5)	36 (63.1)
(iii) Other	**18**	9 (50)	2 (11.1)	0	10 (55.5)
		*P* 0.1	*P* 0.8	*P* 0.3	*P* 0.06
HCC at the time of transplantation					
(i) No	**74**	36 (48.6)	6 (8.1)	0 (0)	52 (70.3)
(ii) Yes	**40**	16 (40)	5 (12.5)	2 (5)	26 (65)
		*P* 0.4	*P* 0.4	*P* 0.06	*P* 0.5
Early liver rejection					
(i) No	**87**	38 (43.7)	9 (10.5)	2 (2.3)	61 (70.1)
(ii) Yes	**27**	14 (51.8)	2 (7.4)	0	17 (62.9)
		*P* 0.4	*P* 0.6	*P* 0.4	*P* 0.4
Time from transplantation					
(i) >3 months–1 year	**11**	6 (54.5)	1 (9.1)	0	8 (72.7)
(ii) >1 year–5 years	**27**	11 (40.7)	5 (18.5)	0	17 (62.9)
(iii) >5 years–10 years	**26**	11 (42.3)	1 (3.8)	2 (7.7)	19 (73.1)
(iv) >10 years	**50**	24 (48)	4 (8)	0	34 (68)
		*P* 0.8	*P* 0.3	*P* 0.09	*P* 0.8
Age group at *T*0					
(i) ≤50 years	**15**	7 (46.6)	1 (6.6)	0	10 (66.6)
(ii) >50–60 years	**34**	13 (38.2)	3 (8.8)	1 (2.9)	27 (79.4)
(iii) >60 years	**65**	32 (49.2)	7 (10.8)	1 (1.5)	41 (63.1)
		*P* 0.6	*P* 0.8	*P* 0.7	*P* 0.2
Therapy at the time of the study					
(i) Cyclosporine	**32**	19 (59.4)	4 (12.5)	0	23 (71.8)
(ii) FK	**55**	26 (47.3)	4 (7.3)	1 (1.8)	36 (65.5)
(iii) Mycophenolate	**12**	4 (33.3)	0 (0)	0	10 (83.4)
(iv) Everolimus	**15**	3 (20)	3 (20)	1 (6.6)	9 (60)
		*P* 0.06	*P* 0.2	*P* 0.4	*P* 0.5

## Abbreviations

ANA: Antinuclear antibodies
ASMA: Anti-smooth muscle antibodies
anti-LKM: Anti-liver-kidney microsomal antibodies
AMA: Antimitochondrial antibodies
OLT: Orthotopic liver transplantation.
